# A comparison of approaches to accessing existing biological and chemical relational databases via SPARQL

**DOI:** 10.1186/s13321-023-00729-5

**Published:** 2023-06-20

**Authors:** Jakub Galgonek, Jiří Vondrášek

**Affiliations:** Institute of Organic Chemistry and Biochemistry of the CAS, Flemingovo náměstí 2, 166 10 Prague 6, Czech Republic

**Keywords:** Resource Description Framework, Relational database, RDB-to-RDF mapping, SPARQL

## Abstract

Current biological and chemical research is increasingly dependent on the reusability of previously acquired data, which typically come from various sources. Consequently, there is a growing need for database systems and databases stored in them to be interoperable with each other. One of the possible solutions to address this issue is to use systems based on Semantic Web technologies, namely on the Resource Description Framework (RDF) to express data and on the SPARQL query language to retrieve the data. Many existing biological and chemical databases are stored in the form of a relational database (RDB). Converting a relational database into the RDF form and storing it in a native RDF database system may not be desirable in many cases. It may be necessary to preserve the original database form, and having two versions of the same data may not be convenient. A solution may be to use a system mapping the relational database to the RDF form. Such a system keeps data in their original relational form and translates incoming SPARQL queries to equivalent SQL queries, which are evaluated by a relational-database system. This review compares different RDB-to-RDF mapping systems with a primary focus on those that can be used free of charge. In addition, it compares different approaches to expressing RDB-to-RDF mappings. The review shows that these systems represent a viable method providing sufficient performance. Their real-life performance is demonstrated on data and queries coming from the neXtProt project.

**Supplementary Information:**

The online version contains supplementary material available at 10.1186/s13321-023-00729-5.

## Introduction

Modern biological and chemical research generates a massive and ever-increasing amount of data originating from various scientific experiments and measurements. For their potential reuse in further research, these data are stored in dedicated databases. A key feature of any database (management) system^1^ is the ability to find required data easily. However, this ability is no longer sufficient in many cases. In many areas of research, it is necessary to combine data from multiple databases. As a result, there is a growing need for databases and database systems to be interoperable with each other. This effort has been supported, for example, by the introduction of the FAIR data principles, which are intended as a guideline to enhance the reusability of data [1].

Many of medium-to-large-scale biological and chemical databases (e.g. PubChem BioAssay [2], ChEMBL [3], Rhea [4] and MolMeDB [5]) are internally stored as relational databases. This approach makes it easy to develop a dedicated server that presents the data and supports data querying. Unfortunately, such a way is usually not very interoperable, and it can be difficult to combine the database with others or to query multiple databases uniformly. To address this gap, some of the databases use Semantic Web technologies, mainly including the Resource Description Framework (RDF) to express their data in an interoperable format [6]. To increase interoperability even more, some of the databases allow data querying by SPARQL, the query language for RDF data [7]. Using these technologies enables the databases to be integrated into the large ecosystem of Semantic Web databases. Such biological and chemical databases include, for example, the protein database UniProt [8], the reaction database Rhea [9], the human-protein database neXtProt [10] and the database of gene-disease associations DisGeNET [11]. Biological and chemical data can also be retrieved through SPARQL from Wikidata [12], where, for instance, the natural product database LOTUS hosts its data [13].

If a database is originally stored as a relational database, there are two basic approaches to make this database accessible through SPARQL. In the first one, the data are exported to an RDF form and stored in a native RDF database system supporting SPARQL querying. The disadvantage of this approach is that either the data are stored twice (and have to be kept synchronised), or a full migration to the native RDF database system is needed (and the original relational database is abandoned). The second approach is to keep the data in the original form and use a system that enables mapping the relational database (RDB) to the (virtual) RDF form. This mapping is used by the system to translate incoming SPARQL queries to equivalent SQL queries, which can be evaluated on the original data by a relational-database system.

Due to the principle on which they work, RDB-to-RDF mapping systems cannot be considered a universal alternative to native RDF database systems. They are merely suitable for certain specific databases for which efficient RDB-to-RDF mappings can be written. Such databases should use only limited sets of predicates as well as small sets of entity classes with a systematic assignment of identifiers to their instances. Another limitation is that SPARQL queries submitted to such RDB-to-RDF mapping systems should only refer to fixed relations between searched entities. Biological and chemical databases usually meet these conditions.

This review examines different currently used RDB-to-RDF mapping systems and various approaches to the design of RDB-to-RDF mappings. It compares them with each other and with the native solutions. The comparison mainly focuses on their application in biological and chemical databases. Our main inspiration for creating this review was that we ourselves develop such an RDB-to-RDF mapping system and we successfully use it to make chemical data available.

In the following sections of this introduction, we briefly describe the basic aspects of the technologies that are relevant to understanding this review.

### Resource Description Framework

RDF has been designed to provide a simple way to make statements about entities, to which it refers as resources. In the RDF data model [6, 14], data are expressed as triples in the form of subject-predicate-object. Each triple expresses a simple statement about its subject, namely that the subject has the property denoted by the predicate and having the value of the object. Resources are referenced by Internationalised Resource Identifiers (IRIs), which makes sure that the resource identifiers have a global meaning. If the global identification of a resource is not important, the resource can be anonymous and represented by a so-called blank node. In such a case, the resource identifier is database-local, and it is defined that the resource cannot be present in any other database. Predicates are also identified by IRIs. From the point of view of RDF, they are resources as well. Therefore, it is possible to make statements about them. The set of predicates used by some database is typically fixed and reflects the data ontologies used. Objects can be either resources or constant values, called literals. Each literal has its value and datatype. Datatypes are denoted by IRIs and they are resources as well. IRIs, blank nodes and literals are collectively referred to as RDF terms.

### Relational database

In contrast, in the relational-database model [15], data are stored in the form of a set of named tables (relations), where each of the tables consists of several named columns (attributes). For each column, the type of the values stored in the column is specified. Such a set of tables and their columns is typically fixed and dedicated for the purpose of a specific database. Individual entries are then stored as rows of the tables. There is no mandatory concept for the description of entities and their identifications. For a given database, the existence of an entity of a certain class is typically expressed by the existence of a record in a table dedicated to this class of entities. One of the table columns (called a primary key) is typically intended to store the entity identifiers. For the identification of entities, integer or string values are mostly used. Unlike RDF, this identification has no global meaning; it is only required that the identifiers be unique for all entities of a given class stored in the database. Other columns of the table can be used to store various properties of the entities. If necessary, it is possible to reference entities from another table by using their primary key values. A column of the table containing these values is called a foreign key.

### RDB-to-RDF mapping

To map a relational database to an RDF database, two closely related steps are required. First, it is necessary to define term mappings between relational-database values and RDF terms and then triple mappings between tables and sets of RDF triples. Each triple mapping defines how a triple is generated from a row of a given source table. It specifies which term mappings and which values from the table row are used to generate individual RDF terms (i.e. subject, predicate and object) of the triple.

For the expression of mappings from relational databases to RDF databases, RDB-to-RDF Mapping Language (R2RML) has been introduced [16]. Although most of current RDB-to-RDF mapping systems support this standard mapping language, they typically also define their own languages, which allow them to take full advantage of their capabilities.

### Querying mapped RDF data

Although a mapping of a relational database to an RDF database can be imagined as a process of generating the RDF database from data stored in the relational database, the RDF database does not need to be explicitly materialised for SPARQL query evaluation. Instead, the mapping is used to translate a SPARQL query into an equivalent SQL query, which is evaluated against the original relational database.

The basic SPARQL language construct is a triple pattern, which is like an RDF triple, but each of its parts may be a variable. The triple pattern matches an RDF data triple if the pattern variables can be replaced by RDF terms for which the resulting triple is equivalent to the RDF data triple. Triple-pattern matching against a queried RDF database produces a solution sequence where each solution maps the variables to the RDF terms for which the resulting triple exists in the database. Further SPARQL construction subsequently works with these solution sequences.

A solution sequence can be represented as a table. The semantics of the other SPARQL constructs is often close to some of SQL constructs. Therefore, the key issue is how to translate pattern matching into a SQL query. The rest of the translation is then relatively straightforward.

The pattern matching against a native RDF database goes through the database triples and checks whether a triple can be matched by a given pattern. In contrast, the pattern matching against a mapped (virtual) RDF database goes through its triple mappings and checks whether a triple mapping can generate triples that are matched by the pattern. If it can, the triple mapping is used to generate the appropriate part of the SQL query that returns a solution sequence in the form of a table.

## Methods

Database systems can be considered from different perspectives. It is possible, for example, to explore their maintainability, extensibility, overall performance, data loading time etc. This review largely focuses on comparing systems by their query performance in a real-life database.

### Selected database systems

The review has been performed on RDB-to-RDF mapping systems and native RDF database systems with a primary focus on those that are used in the fields of biology and chemistry and are available free of charge. Although there are many, mostly historical, approaches, we have limited the selection to only systems that support the current version of SPARQL (i.e. version 1.1) and are thus applicable in the current Semantic Web environment.

The probably most widely utilised RDF database system in the fields of biology and chemistry is Virtuoso [17]. It is used, for example, by UniProt, Rhea, neXtProt, DisGeNET and others. The representation of other RDF database systems is significantly smaller. The Wikidata project employs the Blazegraph database system [18]. BETA [19] (a benchmark for computational drug-target prediction) uses the GraphDB database system [20] for its analysis. GlycoStore [21] (a database of retention properties for glycan analysis) and BioCarian [22] (a search engine for exploratory searches in heterogeneous biological databases) utilise the RDF database system included in the Jena framework [23].

Virtuoso can also operate as a relational-database system and has an extensive support for RDB-to-RDF mappings, which it calls RDF Linked Data View [24]. Another system supporting RDB-to-RDF mappings is Ontop [25]. The main advantage of this system is its support for multiple relational-database systems. In this work, Ontop is specifically tested in connection with two of the most popular opensource relational-database systems - PostgreSQL and MariaDB (a community-developed fork of MySQL). Unfortunately, the disadvantage of this system is that it does not support all SPARQL features. The last RDB-to-RDF mapping system used in this review is the IDSM SPARQL engine, developed by our group [26]. It is based on PostgreSQL and supports all SPARQL features.

Details of the used versions of the selected systems are provided in Additional file 1.

### The selection and preparation of a benchmark database

For the comparison of different approaches of accessing data using SPARQL, we selected the neXtProt human protein database (2021-11-19 release) as a benchmark database. This database is very suitable for our purposes as it has sufficient size (about 1.8 billion triples) and complexity (more than 200 distinct predicates). Moreover, there is a set of real-life SPARQL query examples that can be evaluated against the database. This database is publicly available in the RDF form, but not in the form of a relational database. For this reason, if an RDB-to-RDF mapping system is to be used, the database has to be transformed into the relational-database form.

During the transformation, we follow a few general guidelines. Entities stored in the neXtProt database can be naturally divided into several entity types (e.g. gene, isoform, annotation, evidence, etc.). For each entity type, a dedicated base table is created. Instances (i.e. individual entities) of an entity type are represented by rows of the corresponding base table - each entity is represented by exactly one row. Each base table contains the primary key column storing entity identifiers that are unique for the given entity type. If no entity of a particular type has more than one value of a property, then values of the property can be stored directly in the corresponding base table in a dedicated column. Otherwise, a dedicated property table is created for the given property. This table contains a foreign-key column referencing entities in the corresponding base table and a column storing appropriate property values. It should also be noted that if all instances of an entity type have a property with the same values, these values do not need to be stored directly in the created relational database.

In neXtProt, entities use the following schema of their IRIs in general:

http://nextprot.org/rdf/type/identifier

For a given entity, the identifier part of its IRI can be used directly as an identifier of the entity in the relevant base table and in property tables. The relational database created using this method of entity identification is referred to as the database with direct identifiers.

Most of the neXtProt entity types use string identifiers to identify entities, which is relatively space consuming; in addition, relational-database indexes and joins over string types are not as effective as over integer types. For this reason, we have also created a version that uses integer values to identify all entities.

If an entity uses a string identifier in its IRI, an artificial integer identifier is created and used in all places in the database instead of the original one. To preserve all information, the original value of the identifier is stored in a dedicated column of the relevant base table. This variant of a relational database is referred to as the database with indirect identifiers.

These variants are only general concepts. The particular form of the relational-database schema depends on the relational-database system and the RDB-to-RDF mapping system used. For instance, PostgreSQL supports the single-byte BOOLEAN type, which can be used to store xsd:boolean values from the RDF database. MariaDB uses the single-byte integer type for the same purpose. On the other hand, Virtuoso has no single-byte type that can be used for this, as a result of which it is necessary to use a multibyte integer type.

Other differences arise from the differences between RDB-to-RDF mapping systems. The neXtProt database uses xsd:integer values. For mapping this datatype, the Ontop uses the 8-byte integer type. However, the xsd:integer datatype should represent the infinite set of all integer values. For this reason, the IDSM SPARQL engine uses the variable-length numeric type, which is infinite as well.

To make the selected database more suitable for our purposes, we have slightly modified it. We have corrected the original data files to make them more standard-compliant to achieve the maximum possible compatibility with all RDF database systems. Moreover, in order to improve the results of the transformation, we have added several triples that we considered to be missing. We have reported the missing of these triples to neXtProt maintainers as a issue. We have considered it better to fix these inconsistencies in the database used than to try to capture the same inconsistencies in RDB-to-RDF mapping systems.

### RDB-to-RDF mapping approaches

A way in which a specific relational database is mapped to an RDF database mostly depends on the schema of the source relational database, on the ontology of the target RDF database and on the features of the RDB-to-RDF mapping system used for the mapping. Since the prepared relational databases are created based on the original neXtProt RDF database, RDB-to-RDF mappings from them back to the original RDF form are relatively straightforward and reflect the ways in which the relational-database tables are created from RDF data. The main differences in our RDB-to-RDF mapping approaches arise from the ways in which the SQL values are translated into RDF terms.

For each entity type occurring in neXtProt, it is necessary to define a term mapping between the SQL values that identify the entities in the relational database and the IRIs that identify the corresponding entities in the RDF database. The following descriptions focus mainly on entities that do not use numeric identifiers in their IRIs.

If the database with direct identifiers is used, these term mappings can be simply defined as templates in which specific parts are replaced by SQL values. In our case, SQL values are concatenated with entity type-specific prefixes. We have named the resulting RDB-to-RDF mapping the string approach because both the relational database and the RDB-to-RDF mapping use string values to identify the entities.

In the case of the database with indirect identifiers, the term mappings for entity types using artificial identifiers requires a more complex approach, because artificial integer identifiers cannot be mapped to appropriate IRIs by using simple templates. In this case, a term mapping is specified by a couple of SQL functions, the first of which maps SQL values to IRIs and the other maps IRIs back on SQL values. We named the resulting RDB-to-RDF mapping the integer approach because both the relational database and the RDB-to-RDF mapping use integer values to identify the entities.

However, some RDB-to-RDF mapping systems have very limited possibilities to define term mappings. At its most basic, such a system supports only the template-based approach. In this case, it is still possible to use the database with indirect identifiers if the RDB-to-RDF mapping system supports the specification of triple-mapping source tables as results of complex SQL queries. In this approach, the source tables used in the previous approach are joined (where necessary) by foreign keys with the required base tables to convert artificial identifiers into the original ones. Subsequently, these SQL queries are used as source tables in triple mappings, which can then use the template-based term mappings again. We have named the resulting RDB-to-RDF mapping the combined approach because the relational database and the RDB-to-RDF mapping use different types to identify the entities. The relational database utilises artificial integers to identify the entities, but the RDB-to-RDF mapping employs the original string identifiers.

### Query set

The example queries available at the neXtProt SNORQL page (version as of 1 April 2022) are used as a basis for the benchmark query set utilised in this review [27]. From this set, seven queries using the service statements have been removed as unsuitable for our benchmarking because specific portions of these SPARQL queries have to be invoked against external SPARQL servers. Several queries that use some non-standard features have been modified. We have also modified queries that utilise SPARQL features not supported by Ontop.

Finally, we have checked whether all methods return correct results. Unfortunately, we have encountered many cases where a system returns incorrect results or even crashes. Most of the discovered issues were related to Virtuoso operating as an RDB-to-RDF mapping system. Most issues have been reported (see Additional file 1), and some of them had already been fixed before the final measurement was performed. For others, we try to find workarounds. The workarounds are typically based on modifications of SPARQL queries specifically for the methods affected. Unfortunately, despite our best efforts, one query (NXQ_00058) had to be removed from the query set because it causes crashes of Virtuoso in some scenarios and we had not found any workarounds to fix it.

As a result, the obtained query set contains 140 queries that are correctly evaluated by all the systems tested.

### Benchmark setup

We benchmark all combinations of the selected RDB-to-RDF mapping systems and all designed RDB-to-RDF mapping approaches, with the only exception being Ontop using the integer approach, because the Ontop mapping language cannot express this mapping approach. Therefore, the testing is performed on a total of ten different combinations. For the best performance possible, native mapping languages are used in all the RDB-to-RDF mapping systems tested. In addition to these systems, all the native RDF database systems selected (i.e. Virtuoso, Blazegraph, GraphDB and Jena) are utilised in the benchmark as well. A total of 14 methods of accessing data via SPARQL are thus tested.

For the benchmarks, we use Debian GNU/Linux 11 (Bullseye) running on AMD Ryzen 9 5900X with 64GB RAM and with Samsung 980 PRO 2TB SSD. In all variants, we try to tune the database systems to maximise the utilisation of the hardware used, mainly setting them to take advantage of all the memory.

PostgreSQL and Virtuoso have the support for parallel queries, which means that they allow multiple cores to be used simultaneously to evaluate a single query. Methods using these systems are tested twice - with the support disabled and with the support enabled.

For each method tested, the queries are evaluated one at a time. To minimise the effect of I/O operations, each query is evaluated twice and only the evaluation times of the second run are used.

All relevant sources that are used to set the benchmark are available in the repository accessible online.

## Results and discussion

Before discussing the performance of the methods in real operation, we briefly compare the RDB-to-RDF mapping approaches from the theoretical point of view.

In the combined approach, up to three tables are joined in one triple mapping. This may seem to be highly suboptimal compared to the other approaches, which employ only one table in each triple mapping. However, an RDB-to-RDF mapping system typically has a wide range of different optimisations, so many of these joins can be eliminated. We demonstrate this on several SPARQL-to-SQL translations generated by a hypothetical RDB-to-RDF mapping system.

**Fig. 1:**
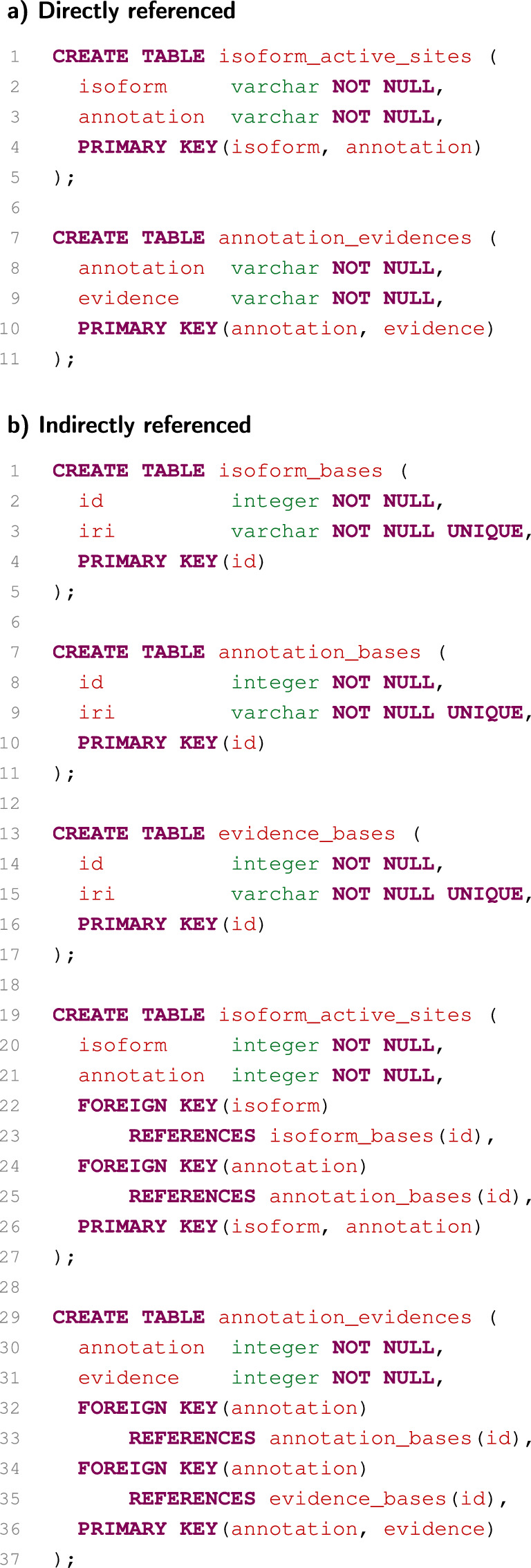
Examples of relational-database schemas

Allow us to consider only a small part of the neXtProt RDF database. The :activeSite property connects isoforms with annotations of active sites. In a relational database, these data are stored in table isoform_active_sites. Similarly, the :evidence property connects annotations with their evidences, and it is stored in table annotation_evidences. If the string approach is used for the RDB-to-RDF mapping, these tables directly contain parts of IRIs identifying the given entities (i.e. the database with direct identifiers is used). The corresponding relational-database schema is depicted in Fig. 1a. If the integer approach or the combined approach is used, the tables contain only artificial identifiers. To translate these artificial identifiers to identifiers used as parts of IRIs, the tables isoform_bases, annotation_bases and evidence_bases are used (i.e. the database with indirect identifiers is used). The corresponding relational-database schema is shown in Fig. 1b.

**Fig. 2:**
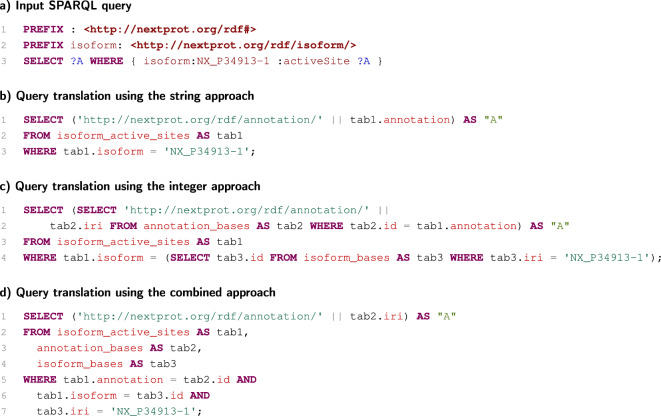
Example of translations of a simple SPARQL query

Now, we assume a simple SPARQL query selecting active-site annotations of a specific isoform (see Fig. 2a). If the string approach is used, the produced SQL code is very simple (Fig. 2b). In the case of the integer approach, the SQL code is also relatively simple, but SQL functions have to be used for term mappings (Fig. 2c). In the example, these functions translating artificial identifiers into IRIs (and back) are expressed as SQL subqueries. If the combined approach is used, the IRIs templates are utilised in the same way as in the string approach. However, three tables have to be joined in that case (Fig. 2d).

Among these three translations of the SPARQL query, the string approach is probably optimal, as all the required data are already available in one table. The combined approach may seem to be the worst in this case, but it is probably comparable to the integer approach. The two approaches generate SQL codes that have to use the same set of tables to obtain solutions. Depending on the relational-database system used, their executing plans can be very similar, especially if the nested-loop join strategy is used.

**Fig. 3:**
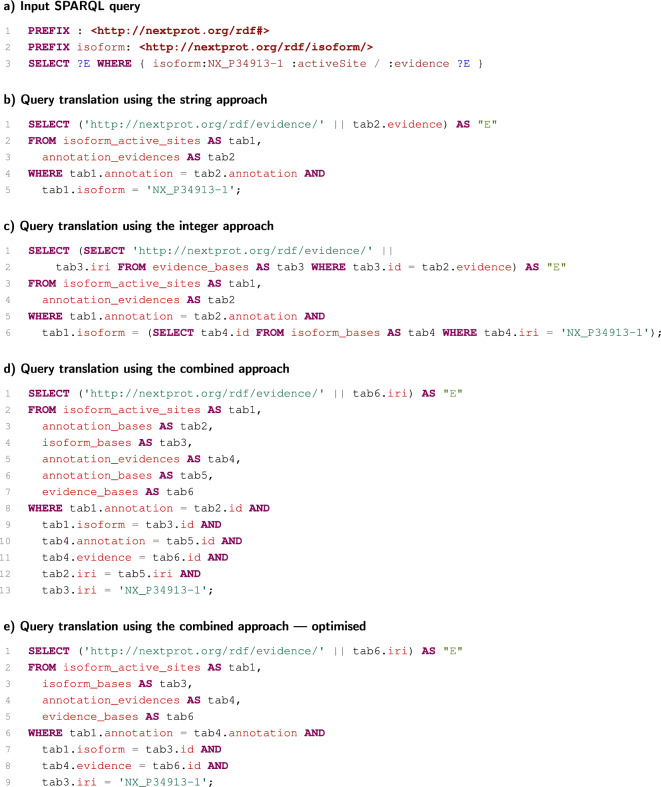
Example of translations of a more complex SPARQL query

The situation is changed when a more complex query is used, which can be demonstrated on a SPARQL query selecting evidences of active-site annotations of a specific isoform (see Fig. 3a).

If the string approach or integer approach is used, the generated SQL codes need only one table join in both cases (Fig. 3b and c). Nevertheless, a join according to string values is not as effective as a join according to integer values in general. In this case, the string approach is probably still the best, but in the case of more complex patterns producing more table joins, the greater overhead of string-based joins overcomes the overhead of the functions translating artificial identifiers.

The situation is different especially if the combined approach is used. If the generated SQL code is not optimised, it is necessary to join six tables in this case (Fig. 3d). This is extremely suboptimal. However, the system can use some key-based optimisations. If a table is joined with itself according to its primary key column, this join can be eliminated. Similarly, if tables are joined according to a foreign key, the join can be eliminated in some cases as well. After these optimisations, the combined approach thus produces the SQL code (Fig. 3e), which is evaluated similarly to the SQL code produced by the integer approach, as it is in the case of the previous SPARQL query.

### Database sizes

Although this is not our primary goal, we measure the spaces required to store the data by the individual database systems. We do not focus directly on the maximum reduction of the necessary space. However, disk I/O operations have a great impact on query performance. Therefore, we try to keep the sizes within reasonable limits.

The size of a database is mainly affected by its storage format. For example, the PostgreSQL database system typically uses a 23-byte row header. In an indirectly referenced database, two integer values having 8 bytes in total are stored in each row of a property table. This means that these 8-byte data are stored in 31-byte rows, which represents a significant overhead in this case.

Nevertheless, the total size may be affected by many settings and various tricks, especially in the case of relational-database systems. In relational databases, we index all columns (except for two long text columns). The fine-tuning of the selection of the columns to be indexed can dramatically reduce size. MariaDB has many possibilities to select a store engine and its row format. It can have a great influence on the database size as well. Nevertheless, we keep the default settings here. In Jena and Blazegraph, disabling quad indexes reduces the space requirements by approximately half.

The sizes of loaded and indexed databases are expressed in Table 1. Due to the differences in individual systems, the sizes are measured only very roughly, yet sufficiently for a basic comparison. Regardless of the relational-database system used, the size of a database with indirect identifiers is significantly smaller than the size of the corresponding database with direct references. Without the need for special tuning, the Virtuoso RDF database is clearly the smallest. Other RDF databases are overcome by relational databases with indirect identifiers. Jena is even surpassed by relational databases with direct identifiers. Overall, in terms of space requirements, it has thus been proved that the relational-database systems used by RDB-to-RDF mapping systems are competitive with native RDF database systems in general.

**Table 1:** The sizes of loaded and indexed databases

Database system	Database size (in GB)		
	Directly referenced	Indirectly referenced	Native RDF
PostgreSQL for IDSM SPARQL engine	123	84	–
PostgreSQL for Ontop	119	80	–
MariaDB for Ontop	117	85	–
Virtuoso	138	96	27
GraphDB	–	–	101
Blazegraph	–	–	106
Jena using TDB2	–	–	239

### Possible sources of bias

Before focusing on the discussion of the performance of the individual methods, it is very important to draw attention to possible sources of bias. Since the neXtProt server from which the query set has been obtained uses Virtuoso, it is to be expected that the query set contains only queries that can be evaluated correctly and in a reasonable time by Virtuoso if it is used as a native RDF database system.

Other possible sources of bias are related to method settings. Although we have tried to configure all the tested methods for the greatest possible performance, it is important to note that our experience with individual methods is very diverse. We have many years of experience running Virtuoso. As authors of the IDSM SPARQL engine, we have deep knowledge of tuning this system. Since the IDSM SPARQL engine is based on PostgreSQL, we also have more experience tuning PostgreSQL than MariaDB.

### Query performance

**Fig. 4:**
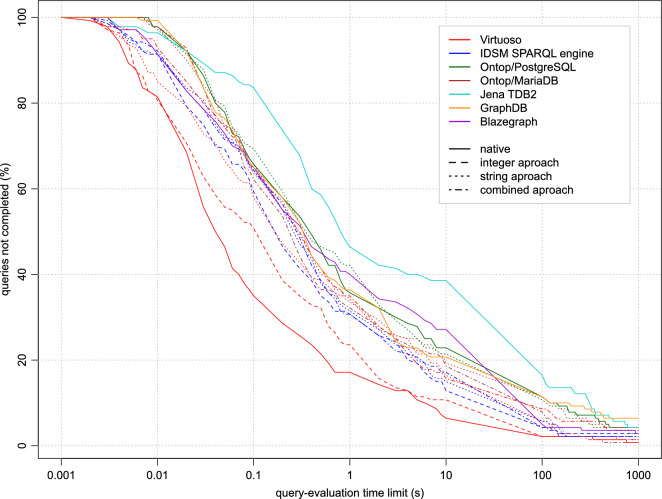
The performance of the methods in the non-parallel query mode

**Fig. 5:**
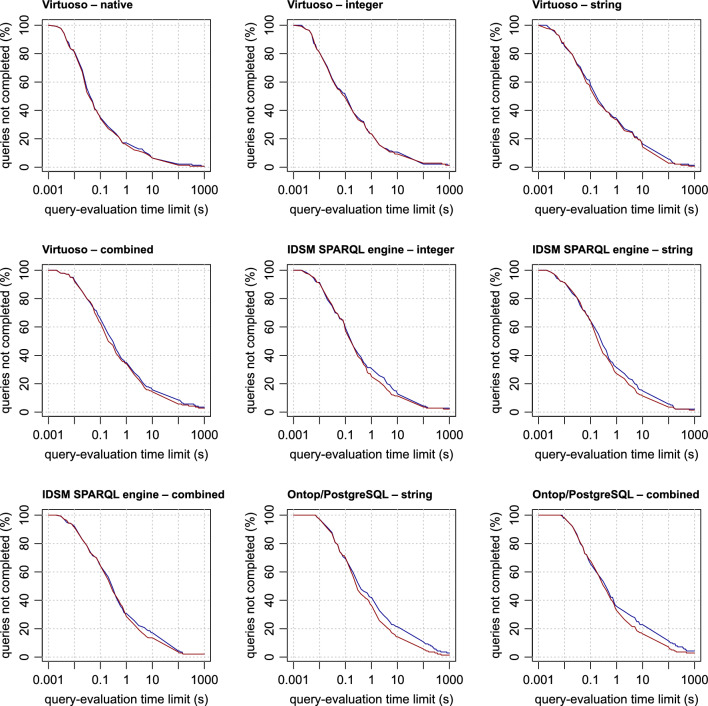
The effect of parallelism. For each method, the red line represents the values measured when the parallel-query support is enabled, the blue line when it is disabled

**Fig. 6:**
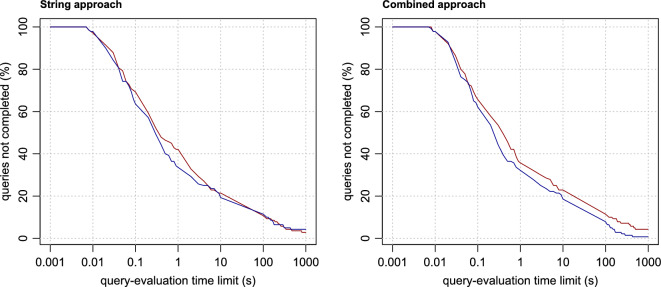
Ontop and the effect of the relational-database system used. For each RDB-to-RDF mapping approach used by Ontop, the red and blue lines represent the values measured for Ontop in combinations with PostgreSQL and MariaDB, respectively

**Fig. 7:**
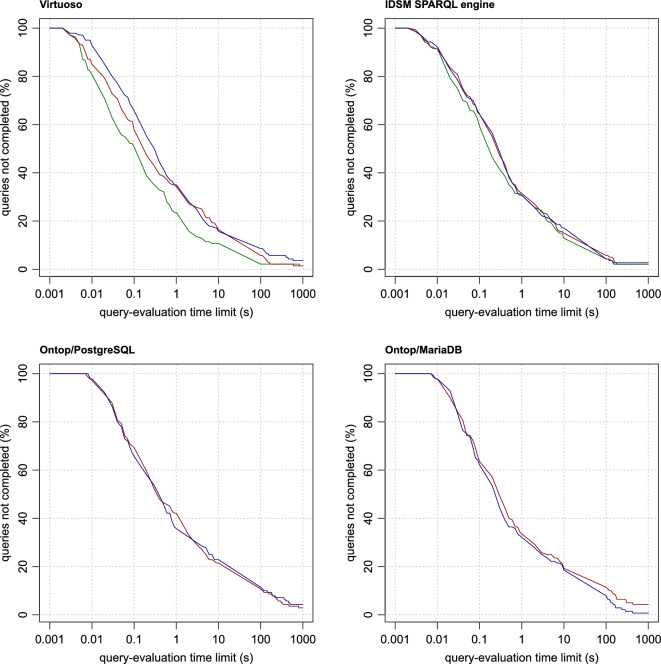
Comparisons of RDB-to-RDF mapping approaches. For each RDB-to-RDF mapping system, the red, green and blue lines represent the values measured for string, integer and combined approaches, respectively

The evaluation time of individual queries varies greatly, and some queries are not even completed within the reasonable time limit at all. For this reason, it does not make sense to compare individual methods based on their average query times. Instead, we use the same method as in our previous work [28]. The query-evaluation times obtained by each of the methods are transformed into plots (Figs. 4, 5 , 6 and 7), where, in each of the plots, the x-axis represents a query-evaluation time limit and the y-axis represents the percentage of the queries not completed within the limit (i.e. the percentage of the queries with query-evaluation times exceeding the limit). It is worth mentioning that it is not necessary to run multiple measurements with different query time limits. All the required values can be derived from a single measurement with a sufficiently large limit. This makes it possible to present the results as continuous plots. It is also important to note that if a method is better than the other tested methods for all query-evaluation limits, it does not imply that all queries are evaluated more quickly by the method than by the others.

When comparing the performance of the methods tested, we mainly focus on the values corresponding to limits exceeding 0.1 s, because anything below this value is probably not very distinguishable by users, especially in situations where the internet-communication overhead must also be taken into account. For a better comparison, the derived values of all the methods for the time limits of 0.1, 1, 10, 100 and 1000 s are summarised in Table 2.

**Table 2:** Method performance

Method			Queries not completed within the time limit (%)				
System	Approach	Parallel	0.1 s	1 s	10 s	100 s	1000 s
Ontop/MariaDB	String	No	63.57	33.57	19.29	11.43	4.29
Ontop/MariaDB	Combined	No	62.14	32.14	18.57	7.86	0.71
Ontop/PostgreSQL	String	No	69.29	42.14	21.43	10.71	2.86
Ontop/PostgreSQL	Combined	No	65.71	35.71	22.86	11.43	4.29
Ontop/PostgreSQL	String	Yes	70.00	36.43	14.29	5.71	1.43
Ontop/PostgreSQL	Combined	Yes	67.86	32.86	16.43	7.14	2.86
IDSM SPARQL engine	String	No	64.29	31.43	15.00	5.71	2.14
IDSM SPARQL engine	Combined	No	64.29	30.71	17.14	4.29	2.14
IDSM SPARQL engine	Integer	No	59.29	30.71	12.86	4.29	2.86
IDSM SPARQL engine	String	Yes	64.29	27.14	11.43	3.57	1.43
IDSM SPARQL engine	Combined	Yes	64.29	28.57	13.57	3.57	2.14
IDSM SPARQL engine	Integer	Yes	57.14	25.00	11.43	3.57	2.14
Virtuoso	String	No	57.86	34.29	16.43	5.71	1.43
Virtuoso	Combined	No	65.71	35.00	15.71	8.57	3.57
Virtuoso	Integer	No	50.71	23.57	10.71	2.14	1.43
Virtuoso	Native	No	35.00	17.14	6.43	2.14	0.71
Virtuoso	String	Yes	55.00	33.57	14.29	2.86	0.71
Virtuoso	Combined	Yes	62.86	34.29	14.29	5.71	2.86
Virtuoso	Integer	Yes	48.57	23.57	9.29	2.86	1.43
Virtuoso	Native	Yes	34.29	15.71	6.43	1.43	0.71
Blazegraph	Native	No	65.00	36.43	20.71	11.43	6.43
GraphDB	Native	No	65.00	40.00	27.14	5.00	2.86
Jena TDB2	Native	No	83.57	46.43	38.57	16.43	4.29

### Query performance in the non-parallel query mode

As shown by the measurements of query-evaluation times obtained for methods in the non-parallel query mode (see Fig. 4), Virtuoso when operated as a native RDF database system is clearly the best method. Among the RDB-to-RDF mapping systems, Virtuoso using the integer approach outperforms the other RDB-to-RDF mapping systems and even all the other native RDF database systems (except for Virtuoso itself). Taken from the other side, the Jena method is outperformed by all methods. The performance of the other methods is much closer to each other. Overall, RDB-to-RDF mapping systems can be competitive with native RDF database systems in general.

### The effect of parallelism

Enabling parallel-query support has a good impact on query-evaluation times in general (Fig. 5). This impact is relatively low in the case of Virtuoso, because Virtuoso uses multiple threads mainly in the parallel computation of hash indexes. In contrast, PostgreSQL makes it possible to split a query-execution plan between multiple worker processes, where every worker process executes a parallel portion of the plan. As demonstrated by the evaluation times measured, this approach makes it possible to achieve much more significant speedup.

### Ontop: PostgreSQL vs. MariaDB

Ontop achieves good performance with both relational-database systems tested (Fig. 6). It follows from the measured evaluation times that it has better performance in combination with MariaDB, especially if the combined approach is used. Since Ontop generates essentially identical SQL queries for both systems, the main differences in the times measured are probably caused by differences in the ways that these relational-database systems use to evaluate the SQL queries.

### A comparison of RDB-to-RDF mapping approaches

The measured data can be used to infer general performance characteristics of the RDB-to-RDF mapping approaches (Fig. 7). In general, the integer approach has better performance than the string approach on the same RDB-to-RDF mapping system. The performance of the combined approach is comparable with the performance of the string approach in the cases of the IDSM SPARQL engine and Ontop/PostgreSQL, or even slightly better in the case of Ontop/MariaDB. In Virtuoso, the combined approach is outperformed by the other two approaches. However, it is probably a consequence of the fact that Virtuoso does not perform all the join optimisations described above. In general, the integer approach seems to be the best choice. If an RDB-to-RDF mapping system does not allow the use of this approach, then the combined approach seems to be a good choice, because its performance is comparable to the string approach, but its storage requirements are lower.

## Conclusion

The review shows that RDB-to-RDF mapping systems can be useful for making the existing biological and chemical databases available via SPARQL. It demonstrates that these systems have sufficient performance to evaluate complex queries in a non-trivially large life-science database. Some of them have even outperformed some of native RDF database systems.

The best results have been achieved by Virtuoso. This database system outperforms the other approaches regardless of whether it is used as a native RDF database system or as an RDB-to-RDF mapping system. Unfortunately, while working with this system, we encountered the highest number of issues, especially when it was used as an RDB-to-RDF mapping system. We were able to find workarounds for most of these issues. Nevertheless, it would have been nearly impossible to identify these issues in many cases if we had not had the results of other methods, which we could use for comparison.

The performance of the other two tested RDB-to-RDF mapping systems is comparable. Ontop supports more relational-database systems, but, unfortunately, it does not support all features of the SPARQL language. In addition, it is more user-friendly, because it uses a simple and easy-to-use RDB-to-RDF mapping language to describe RDB-to-RDF mappings. The disadvantage of this language, however, is that it is not as expressive as other languages, and it does not make it possible to express all the mapping approaches tested.

The IDSM SPARQL engine fully supports the SPARQL standard, but it is closely tied to PostgreSQL and cannot be used with other relational-database systems. Currently, it does not use any RDB-to-RDF mapping language, and RDB-to-RDF mappings have to be constructed directly from the Java code. This is not very user-friendly; on the other hand, it is a very flexible solution, because it allows users to extend the engine and thereby directly influence the SQL code produced.

The comparison of different approaches to RDB-to-RDF mappings demonstrates that it is better to utilise approaches based on the use of artificial identifiers. In general, the performance of these approaches is typically better, and the database also has lower space requirements in such cases. Of course, in a real-life situation, a relational database is typically already created, and it is thus not directly tailored to a particular RDB-to-RDF mapping approach. However, relational databases themselves typically already use suitable identifiers to achieve good SQL-query performance and low space requirements. Moreover, the comparison shows that to keep good performance, it is not necessary to utilise these identifiers directly as parts of constructed IRIs that are used to identify entities. Consequently, it is typically possible to design nice user-friendly IRIs without a significantly negative impact on performance. Therefore, when using such a real-life database, the performance of a created RDB-to-RDF mapping should be comparable with the approaches using relational databases with indirect identifiers.

## Supplementary Information

**Additional file 1: **Details of the used versions of the selected systems.

## Data Availability

The benchmark database used for the comparison is openly available on Zenodo (https://doi.org/10.5281/zenodo.7071135). Other resources used for the comparison are available in the GitHub repository (https://github.com/galgonek/r2rms-review).

## Footnotes

1 The term ‘database system’ is often abbreviated simply to ‘database’. In order to prevent confusion, this abbreviation is never used in this text, and the term ‘database’ always refers to its original meaning, i.e. an organised collection of related data.
