# Supplementary material for: A comparison of approaches to accessing existing biological and chemical relational databases via SPARQL
Source: J Cheminform. 2023 Jun 20;15:61. doi: 10.1186/s13321-023-00729-5 (PMC10280967; doi:10.1186/s13321-023-00729-5)
Supplement: Supplementary file 1 — Additional file 1: Details of the used versions of the selected systems. [file 13321_2023_729_MOESM1_ESM.pdf]

Additional file 1 – The software used

Blazegraph

Website: https://blazegraph.com
Version: 2.1.6

GraphDB

Website: https://www.ontotext.com
Version: 10.0.0 (Free Edition)
Issues found:

- *Incorrect translations of object lists combined with property paths.* It is related to a third-party library used and it is worked around by rewriting the affected queries not to use this syntax. See https://github.com/eclipse/rdf4j/issues/3220.

Jena

Website: https://jena.apache.org
Version: 4.5.0
Issues found:

- *SPARQL query comparing `count(distinct ?v)` values is evaluated incorrectly.* It is worked around by rewriting the affected queries not to use this syntax. See https://issues.apache.org/jira/projects/JENA/issues/JENA-2342.

Virtuoso

Website: https://virtuoso.openlinksw.com
Version: 7.2.7 (Open-Source Edition), patched
Issues found:

- *Wrong behaviour of SQL `WHERE`.* It is already fixed in the version used. See https://github.com/openlink/virtuoso-opensource/issues/974.
- *Variable in `T_IN` list and the corresponding variable in `T_OUT` get values from columns that are too different.* It is worked around by rewriting the affected queries not to use this syntax. See https://github.com/openlink/virtuoso-opensource/issues/975.
- *Internal error: `ssg_print_box_as_sql_atom()`: bad mode.* It is worked around by rewriting the affected queries to use explicit iri function calls. See https://github.com/openlink/virtuoso-opensource/issues/976.
- *Missing `IS NOT NULL` condition.* It is worked around by adding explicit not-null checks to RDB-to-RDF mappings. See https://github.com/openlink/virtuoso-opensource/issues/977.
- *Incorrect `TOP 1` optimisation.* It is worked around by a patch disabling the wrong optimisation. See https://github.com/openlink/virtuoso-opensource/issues/978.
- *Wrong behaviour of SPARQL `DISTINCT`.* It is worked around by enclosing the affected queries into subqueries. See https://github.com/openlink/virtuoso-opensource/issues/979.
- *Some SPARQL queries are not evaluated correctly if the `xsd:string` datatype is present.* It is worked around by rewriting the affected queries not to use the `xsd:string` datatype explicitly. See https://github.com/openlink/virtuoso-opensource/issues/1064.
- *Specific expressions in filter conditions cause crashes of Virtuoso.* It is fixed by a patch backported from the devel branch. See https://github.com/openlink/virtuoso-opensource/issues/1065.
- *Wrong behaviour related to the use of indexes with the `UNIQUE` attribute.* When the combined approach is tested, the use of the attribute causes that the results of several queries contain entities that should not be included in them. To work around this issue, the attribute has been omitted.
- *Wrong behaviour related to the use of hash joins.* Further issues that make the query evaluation fail or the server crash have been worked around with the appropriate addition of the `LOOP` table option.

Ontop

Website: https://ontop-vkg.org
Version: 4.2.1
Issues found:

- *Variables only used in `MINUS` should not be in-scope in the query.* It is already fixed in the version used. See https://github.com/ontop/ontop/issues/447.
- *The `substr` function is not defined for bigint arguments.* It is worked around by adding the missing SQL stored procedures. See https://github.com/ontop/ontop/issues/545.
- *Incorrect translations of object lists combined with property paths.* The issue is related to a third-party library used and it is worked around by rewriting the affected queries not to use this syntax. See https://github.com/eclipse/rdf4j/issues/3220.

IDSM SPARQL Engine

Website: https://idsm.elixir-czech.cz
Version: c49d9337 (git)
Issues found:

- The issues were fixed internally during the development as the neXtProt database had been used for testing.

PostgreSQL

Website: https://www.postgresql.org
Version: 14.4

MariaDB

Website: https://mariadb.com
Version: 10.6.8
